# Experimental Study on Cementless PET Mortar with Marble Powder and Iron Slag as an Aggregate

**DOI:** 10.3390/ma16155267

**Published:** 2023-07-27

**Authors:** Shahid Ullah Khan, Abdur Rahim, Nur Izzi Md Yusoff, Ammad Hassan Khan, Saadia Tabassum

**Affiliations:** 1 Department of Transportation and Management, University of Engineering and Technology, Lahore 54890, Pakistan; shahidkhann46@gmail.com (S.U.K.); chair-tem@uet.edu.pk (A.H.K.); saadia@uet.edu.pk (S.T.); 2 Department of Civil Engineering, Universiti Kebangsaan Malaysia, Bangi 43600, Malaysia

**Keywords:** concrete, durability, iron slag, pet binder, plastic concrete, waste marble

## Abstract

There has been an increase in plastic production during the past decades, yet the recycling of plastic remains relatively low. Incorporating plastic in concrete can mitigate environmental pollution. The use of waste polyethylene terephthalate (PET) bottles as an aggregate weakens properties of concrete. An alternative is to use PET bottles as a binder in the mortar. The PET binder mixed with sand results in weak mortar. Marble and iron slag can enhance PET mortar properties by preventing alkali reactions. This study examines the mechanical and durability properties of PET mortar with different mixes. The mixes were prepared as plastic and marble (PM); plastic and iron slag (PI); plastic, sand, and marble (PSM); plastic, iron slag, and marble (PIM); and plastic, sand, and iron slag (PSI). PM with 30–45% plastic content had increased compressive and flexural strength up to 35.73% and 20.21%, respectively. PI with 30–35% plastic content showed strength improvements up to 29.19% and 5.02%, respectively. However, at 45% plastic content, strength decreased by 8.8% and 27.90%. PSM, PIM, and PSI specimens had nearly double the strength of ordinary Portland cement (OPC) mortar. The durability of PET mortar in chemical solutions, mainly 5% HCl and 20% NaOH, indicate that mass decreased after 3, 7, and 28 days. All specimens showed good resistance to HCl and NaCl solutions compared to OPC mortar. However, its resistance to NaOH is low compared to OPC mortar. PET mortar without cement showed higher strength and durability than cement mortar, making it suitable for paver tiles, drainage systems, and roads.

## 1. Introduction

Plastic waste is on the rise, with 400 million tons produced annually and predicted to reach 1.1 billion by 2050. Only 10% of the 7 billion tons generated have ever been recycled [1]. PET plastic makes up 82 million tons of annual plastic production [2]. In the construction industry, cement production is associated with the unsustainable use of natural resources, leading to economic and environmental challenges [3,4]. PET plastic has the potential to serve as a viable alternative to cement in the preparation of concrete and mortar, providing a means to mitigate the adverse effects of environmental pollution and natural resource depletion.

There are different ways of using waste PET in concrete. One method is the use of PET fine aggregates in concrete to form PET concrete [5]. The second method involves using PET in the form of fibres in concrete [6,7,8]. The third method is to use PET as a binder in concrete [9,10]. Many researchers used PET plastic bottles in concrete as an aggregate; however, strength reduces with increasing plastic content [11,12,13]. The riverbed soil replaced with waste PET as an aggregate had decreased compressive and splitting tensile strength of 6% and 19%, respectively [14]. Ismail and AL-Hashmi (2008) partially substitute sand with plastic in concrete and found that waste plastic reduces the strength of concrete due to its hydrophobic nature [15]. Plastic as a fine aggregate decreases adhesive strength and slows down the cement hydration process due to its smooth surface, resulting in a poor bond and slipping during compression testing. Pezzi et al. (2006) found that the compressive and flexural strength decreases by increasing PET particles of size 15–25 mm diameter as a coarse aggregate in PET concrete [16]. Marzouk et al. (2007) also investigated the use of PET bottles as substitutes for sand in concrete. The study found that replacing sand (ranging from 0 to 100%) with PET resulted in reduced compressive and flexural strength in the concrete composites [17]. Adeboje et al. (2020) investigated the effects of replacing cement with bentonite clay and sand with crumb rubber simultaneously on the engineering properties of concrete. The optimum compressive and tensile strength of concrete was achieved via the substitution of 0.5% cement and 0.5% sand with bentonite clay and crumb rubber, respectively [18]. Jassim (2017) investigated the replacement of sand with the addition of recycled plastic to cement. The study showed that plastic cement reduced strength and density but improved workability and ductility [19]. Al-manaseer and Dalal (1997) discovered that using 10, 20, and 30% plastic as an aggregate reduced compressive strength up to 34, 51, and 67%, respectively [20]. Gao et al. (2021) used coal gangue as a replacement for coarse aggregate in structural concrete, which offers environmental benefits like waste management, conserving natural resources, reducing energy consumption, and lowering the carbon footprint of concrete production [21]. This highlights the use of waste materials for economic and environmental benefits. Choi et al. (2009) made waste PET lightweight aggregates (WPLA) using a shredded PET bottle (5–15 mm) and mixing it with sand powder (passing through a 0.15 mm sieve) at 250 °C. The compressive strength of mortar prepared from cement, WPLA, and river sand decreased by increasing WPLA content from 25 to 75% [22]. Fibre-reinforced concrete helps in crack reduction; however, the strength was reduced due poor bond between the plastic fibre and cement [23,24,25].

Plastic mortar having unsaturated resin of PET as a binder has higher compressive and flexural strength. The strength increases by increasing PET resin in PET mortar [26]. Gao et al. (2019) conducted a comprehensive review of unsaturated polyester concrete (UPC) and found that UPC requires controlling resin content (10–20%) for mechanical properties. UPC offers potential for waste utilisation, but humidity, temperature, and ageing must be managed. Chemical resistance is good, except in extreme environments. The fillers reduce shrinkage and improve creep resistance, fibre enhances toughness and impact resistance, and grafting improves fibre–matrix interactions. PET synthesis into UPC promotes sustainability, but curing optimisation and cost reduction are important areas for future study [10]. The energy required to convert recycled PET to unsaturated resin can be saved if waste PET bottles are directly used in concrete. Thiam et al. (2021) used melted high-density polyethylene (HDPE) and low-density polyethylene (LDPE) to synthesise four plastic contents (45, 50, 60, and 65%) that were mixed with sand and HDPE/LDPE blends in different weight ratios (40/60, 50/50, and 60/40) were used. A blend ratio of 50/50 in samples with plastic contents of 50% and 60% showed that the highest compression strength can be achieved but still less than OPC mortar [27]. Kumi-Larbi et al. (2018) produced LDPE-bonded sand blocks from melted waste LDPE and water sachets and mixed them with sand. The density, compressive strength, and water adsorption as a function of sand particle size and sand-to-plastic ratio were studied and found that the maximum compressive strength of 27 MPa can be achieved [28]. Ge et al. (2013) used molten PET plastic and examined that strength increased by increasing sand to plastic content ratio. The compressive strength of PET mortar increased by 56.6%, which was 35.7 MPa when the sand-to-PET ratio was increased from 1:1 to 4:1 [29]. Ge et al. (2015) also examined a PET plastic mortar made with clay brick, which had compressive and flexural strengths that were 42.5 and 12.6 MPa, respectively [30]. These optimum strengths were achieved at PET to clay brick ratio of 1:2. 

Using marble and iron slag as a fine aggregate instead of sand can enhance the strength and durability of plastic mortar made from melted PET and aggregates. Rasheed et al. (2022) made sulphur concrete using waste marble powder (WMP), river sand (RS), and GGBS. SC-GGBS had good resistance to NaOH, and SC-RS, SC-WMP, and SC-GGBS had less mass loss in NaCl solution than that of OPC mortar [31]. This research includes the utilisation of marble waste and iron slag with waste PET bottles directly (without converting it to unsaturated polyester resin) to produce an economical and sustainable PET mortar as an alternative OPC mortar. The strength improvements need investigations for optimising the plastic content in PET mortar. Marble and iron slag used as an aggregate contain less alkali than cement and sand, so there is a need to investigate its expected higher resistance to harsh chemical environments like sodium hydroxide (NaOH), hydrochloric acid (HCl) and sodium chloride salt (NaCl) solutions. It is also needed to evaluate the recyclability of PET mortar for its use as a sustainable alternative to cement mortar. The effective use of PET plastic in mortar as a complete cement replacement can be useful for manufacturing paver tiles, concrete blocks, toxic waste containers, and drainage purposes. 

## 2. Materials and Methods

### 2.1. Materials

**Figure 1:**
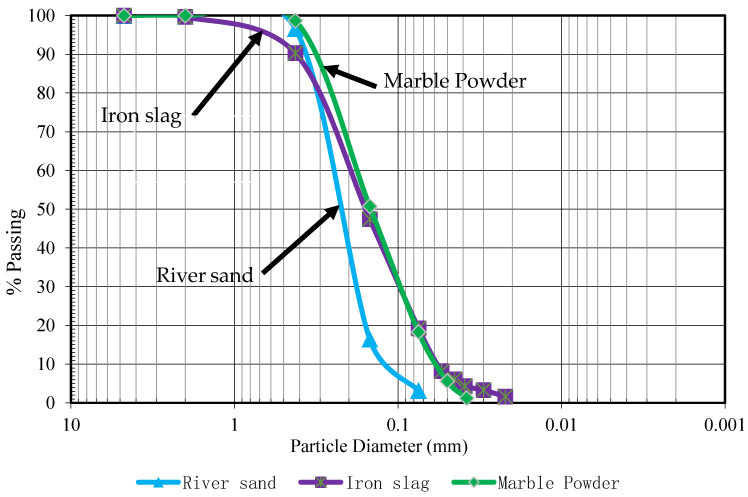
Gradation curve for river sand, iron slag, and marble powder.

The waste PET was obtained from beverage and mineral water bottles after removing the bottle cap and label. The bottles were then washed and cleaned of residues and dust using tap water. After drying in sunlight for 48 h, the bottles were shredded into small pieces and melted down at 280 °C at the time of concrete sample preparation. River sand, waste marble, and iron slag were used as aggregate in PET mortar. These fine aggregates were used in different proportions. The iron slag was obtained as a by-product from the blast furnace, and waste marble powder was procured from a marble manufacturing factory. According to Unified Soil Classification System (USCS), all these fine aggregates were poorly graded. Figure 1 shows the particle size distribution of river sand, waste marble powder, and iron slag as per ASTM D6913 (2004) [32]. Table 1 shows the physical properties of these aggregates, and the elemental composition of river sand, marble powder, and iron slag is given in Table 2.

### 2.2. Mix Design

The specimens prepared were two and three ingredient mixes. The two ingredient mixes were waste PET plastic mixed with marble powder (PM specimens) and waste PET plastic mixed with iron slag (PI specimens). In these specimens, PET plastic content was increased from 25 to 45% in an incremental proportion of 5%. Three ingredient mixes included PET, sand, and marble (PSM specimens); PET, iron slag, and marble (PIM specimens); and PET, sand, and iron slag (PSI specimens). The control sample of ordinary portland cement (OPC) mortar with a cement and river sand ratio of 1:2.54 was prepared using tap water and was cured for 28 days in tap water. Table 3 shows different mixed-design proportions.

### 2.3. Specimen Preparation

**Figure 2:**
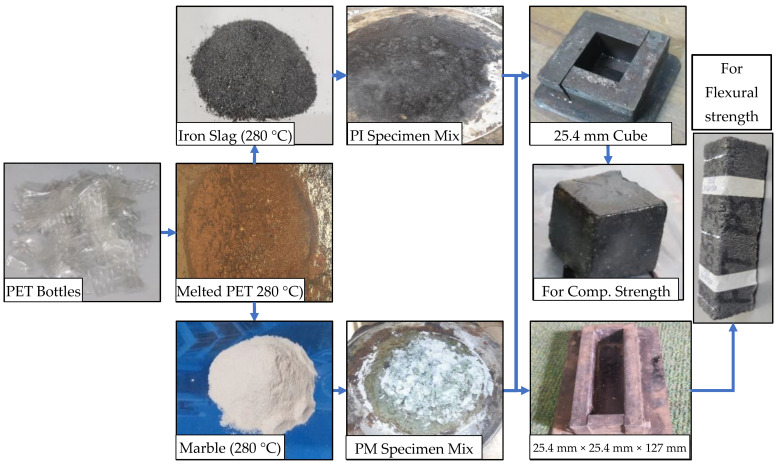
PM and PI specimen preparation.

Specimens of PET concrete were prepared according to ASTM C348 and ASTM C349 [34,35]. The shredded waste PET bottles were melted at a temperature of 280 °C. The aggregates were preheated in the oven at a temperature of 280 °C for 4 h. The 280 °C melted PET plastic was then mixed with aggregates in various proportions to form PET mortar and poured into 280 °C preheated mould. The PET mortar in the mould was compressed immediately after pour and was allowed to cool at room temperature for 3 h in the mould. The size of the mould was 25.4 mm cube and 25.4 mm × 25.4 mm × 127 mm to cast samples for compressive and flexural strength tests, respectively. Figure 2 shows the plastic–marble and plastic–iron slag mix preparation at 280 °C. 

### 2.4. Test Methods

**Figure 3:**
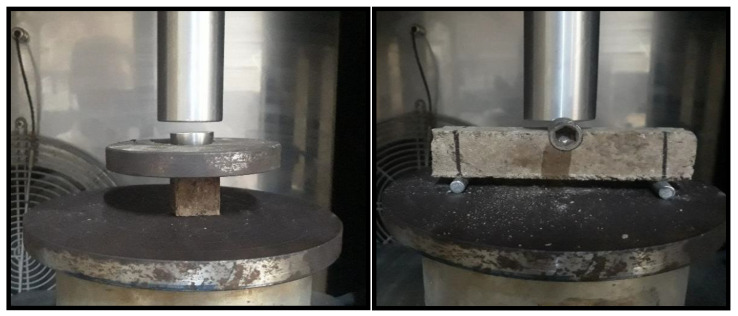
Compressive and flexural strength test on plastic mortar.

**Figure 4:**
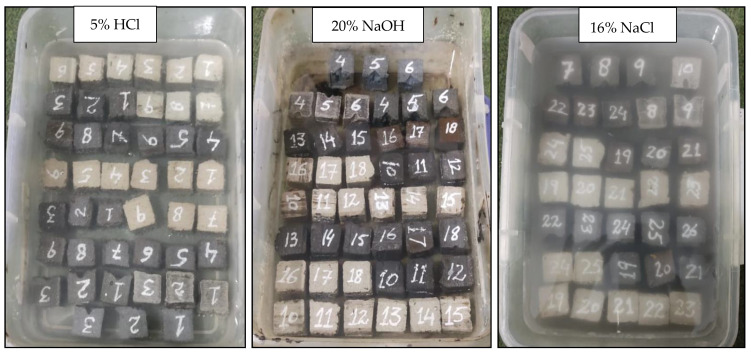
Mortar specimen with identification numbers placed in chemical environments.

The PET mortar specimens were tested in the universal testing machine with a loading rate of 5 mm/min. The compressive and flexural strengths were found according to ASTM C349 and ASTM C348, respectively. The experiment involved testing three specimens for each mortar composition. The average compressive and flexural strength values were obtained for each mortar composition. The setup for compressive and flexural tests is shown in Figure 3. For durability, solutions of 5% hydrochloric acid (HCl), 20% sodium hydroxide (NaOH), and 16% sodium chloride (NaCl) were prepared. PM, PI, PSM, PIM, and PSI plastic mortar cubes of 25.4 mm cubes were dried for 24 h and immersed in these solutions for 3, 7, and 28 days. After each interval of time, the specimens were oven dried, and mass loss was determined. The chemical solutions were replaced with a fresh solution of the same concentration every week to maintain the durability environment. The specimens immersed in the chemical solution are shown in Figure 4. After 28 days, samples were tested for compressive strength loss. Scanning electron microscopy with energy-dispersive X-ray spectroscopy (SEM with EDX) was used to find out elemental composition of waste marble powder, river sand, and iron slag. 

## 3. Results and Discussion

### 3.1. Effect of PET Content on Compressive Strength Behaviour

**Figure 5:**
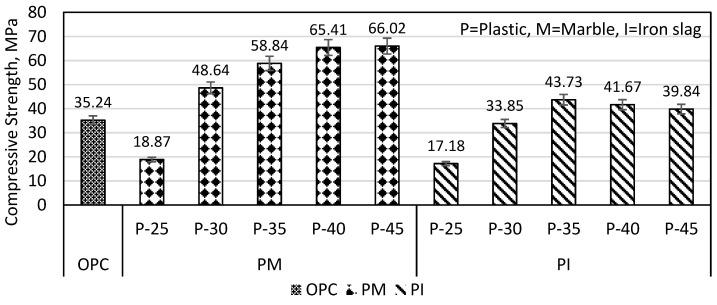
Effect of PET content on PM and PI compressive strength.

The compressive strength of plastic mortars made of plastic and marble (PM specimens) is shown in Figure 5. The results show that by increasing plastic content from 30 to 45%, the compressive strength increased from 48.64 to 66.02 MPa, which was 35.73%. The compressive strength of the PM specimen becomes steady at 40–45% plastic content.

Rebeiz et al. (1996) used unsaturated resin (obtained from waste PET) and found that the strength and durability of polyester concrete are higher than ordinary portland cement [36]. Marble particles were fine and required more plastic to bind particles with each other and required more time to blend the mixture completely.

**Figure 6:**
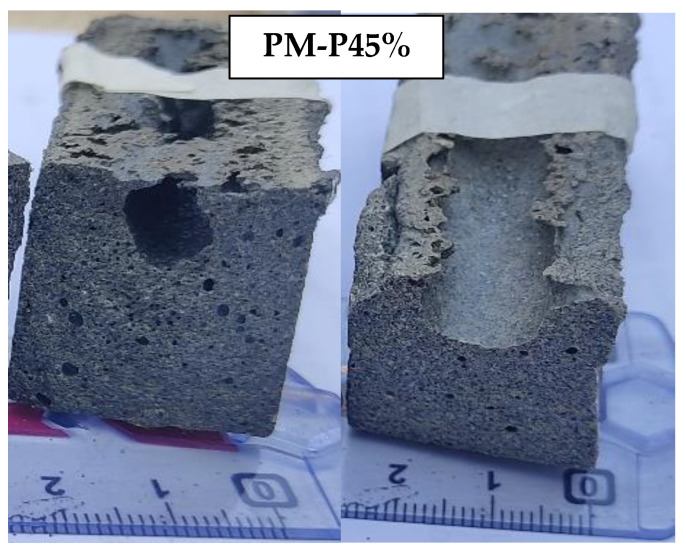
PM specimens with 45% plastic.

Plastic and iron slag (PI specimen) results showed that by increasing plastic content from 30 to 35%, the compressive strength increased from 33.84 to 43.72 MPa which was 29.19%, but when plastic further increased to 45%, the compressive strength decreased from 43.72 to 39.83 MPa (8.8% decrease). The optimum strength was also achieved by Ge et al. (2015) at a PET content of 33.33% with recycled clay brick [30]. During the preparation of PM and PI specimen, it was observed that a plastic content of less than 25% was inadequate to establish the bond between the aggregates completely. The presence of excessive plastic content (45%) in a molten state in PM and PI specimens hinders the escape of hot air during the compaction process. As a result, large size voids are made as shown in Figure 6. Based on the compressive strength consideration, the optimum plastic content was selected as 40% and 35% for PM and PI specimens, respectively. Moreover, at every percentage of plastic content, the PM specimen had higher compressive strength than that of the PI specimen, indicating a good bond of plastic with marble than iron slag.

### 3.2. Sand, Marble, and Iron Slag Substitution Impact on PET Mortar Strength

**Figure 7:**
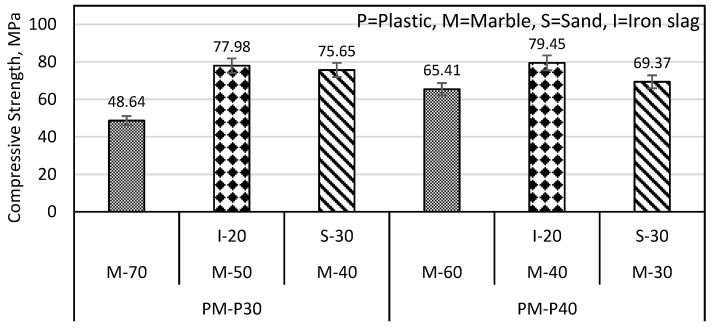
Sand and iron effect on PM sample compressive strength.

In Figure 5, the PM-P30% specimen had a compressive strength of 48.64 MPa, which could be increased by substituting marble either with iron slag or sand. According to Figure 7, the PM-P30% specimen had a compressive strength of 48.64 MPa, which could be increased by 60.32% when marble was substituted with iron slag, i.e., 50% marble and 20% iron slag (M-50 specimen). Similarly, the PM-P30% specimen compressive strength increased up to 55.53% by substituting only 30% marble with sand. The SEM images in Section 3.3 showed that the iron slag and sand particles are coarser and more angular than marble particles. These particles had lesser surface area and more available binder, therefore, resulting in increased compressive strength.

Similarly, from Figure 5, it can be observed that the compressive strength of the PM-P40% specimen is 65.41 MPa, which is attributed to substituting marble either with iron slag or sand. In Figure 7, the PM-P40% specimens had a compressive strength of 65.41 MPa, which could be increased up to 21.46% by substituting marble with iron slag, i.e., 40% marble and 20% iron slag. In the case of replacing 30% marble with 30% sand in PM-P40%, there was a slight increase from 65.41 to 69.37 MPa (i.e., 6.05%) in compressive strength.

**Figure 8:**
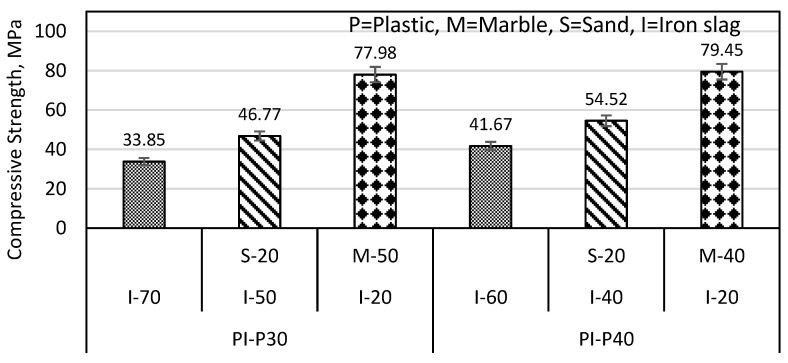
Sand and marble effect on PI sample compressive strength.

The PI-P30% specimen has a compressive strength of 33.85 MPa, as shown in Figure 5. Figure 8 shows that the compressive strength of the PI-P30% specimen, which is 33.85 MPa, can be increased by 38.16% (i.e., 46.77 MPa), if 20% of iron slag is replaced by sand and by 130.3% (i.e., 77.98 MPa) when 50% of iron slag was replaced with marble. In the case of PI-P40% specimens, compressive strength improved up to 30.83% (i.e., 54.52 MPa) if 20% iron slag was replaced with sand and improved up to 90.66% (i.e., 79.45 MPa) if 40% iron slag was replaced with marble. From the results of Figure 7 and Figure 8, it was concluded that the strength improvement was less in the plastic mortar with a higher amount of plastic. 

### 3.3. Impact of Ingredient Variation on Plastic Mortar Strength

**Figure 9:**
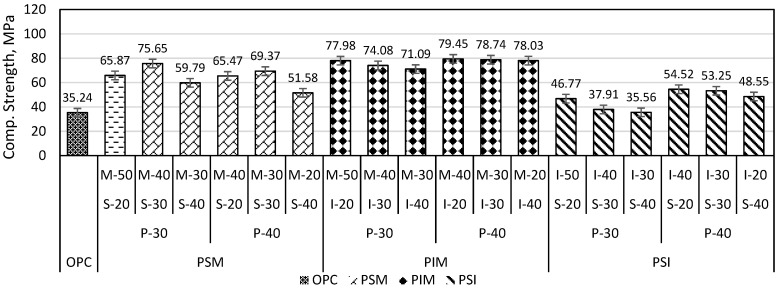
Compressive strength of plastic mortar (with 3 ingredients).

Plastic mortars with three ingredients were PSM (plastic, sand, and marble), PIM (plastic, iron slag, and marble), and PSI (plastic, sand, and iron slag). In Figure 9, the compressive strength of the PSM-P30% and PSM-P40% specimens increased from 65.87 to 75.65 MPa (i.e., 14.84%) and from 65.47 to 69.37 MPa (i.e., 5.95%), respectively, when the sand content was increased from 20% to 30%. However, if the sand content was further increased to 40% in the PSM-P30% and PSM-P40% specimens, the strength decreased to 59.75 (i.e., 26.52%) and 51.58 MPa (i.e., 34.49%), respectively, as shown in Figure 9. Ge et al. (2015) used PET as a binder and clay brick waste as an aggregate to form PET mortar. The compressive and flexural strength increases when the binder-to-clay brick ratio increases from 1:1 to 1:2, but if it further increased to 1:3, the strength is reduced [30]. PSM optimum compressive strength achieved at mix proportion (30% plastic, 30% sand, and 40% marble) was 2.14 times higher than that of OPC mortar. The optimum percentage (30% plastic) in the PSM specimen is in agreement with the results of Ge et al. (2013) [29]. 

**Figure 10:**
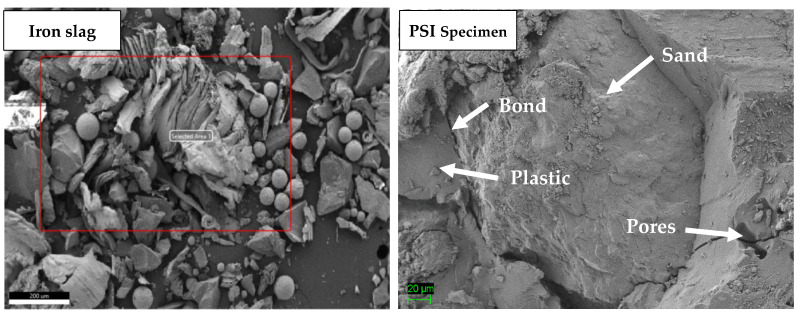
Iron slag and PSI specimen SEM images.

**Figure 11:**
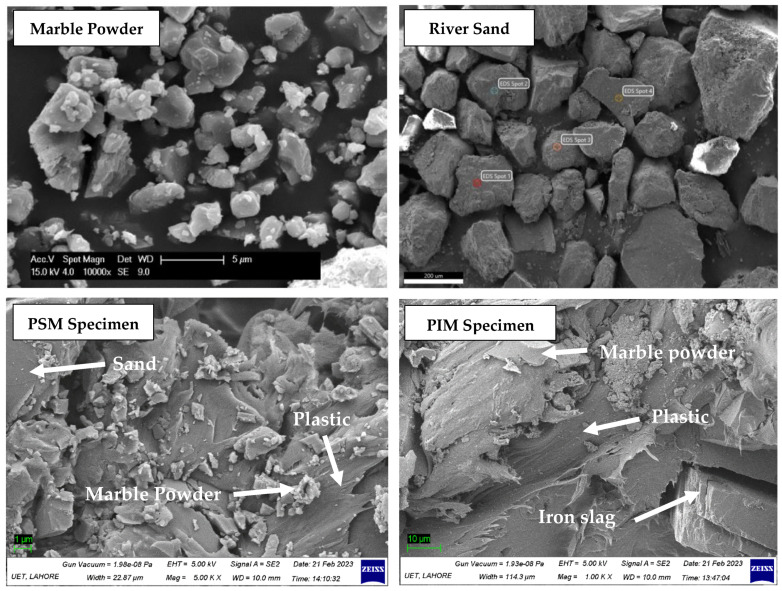
Marble, sand, PSM, and PIM specimen SEM images.

PIM specimens were the mixture having the highest strength among all mortar composites. PIM specimen strength decreased by increasing iron slag, as shown in Figure 9. The optimum strength of PIM was 2.25 times greater than that of OPC mortar. The SEM image of iron slag and PIM is shown in Figure 10. It showed that iron slag had both angular as well as round particles, and marble had uniform particle size with angular texture. The higher percentage of iron slag required a larger amount of plastic to bind with each other. Also, the PIM specimen SEM image, as shown in Figure 11, shows that iron slag particles in the PIM specimen had no perfect bond with plastic compared to marble powder.

When marble powder in PSM specimens is completely replaced by iron slag, the strength is further reduced, and these mixtures are denoted by PSI. Figure 9 shows that the compressive strength of PSI specimens decreased with increasing sand from 20 to 40%. Shaaban et al. (2021) found that replacing 10% sand with crumb rubber reduces the compressive strength of concrete [37]. Noui et al. (2020) also found that increasing iron slag and decreasing sand in OPC mortar led to higher compressive strength [38]. Similarly, Humam and Siddique (2013) found that increasing iron slag content and decreasing sand content increases the strength of cement mortar [39]. The scanning electron image (SEM) of the PSM specimen in Figure 11 showed that sand had a weaker bond with plastic compared to marble particles. This weaker bond decreased strength further when sand content increased from 30 to 40%. PSI mix proportion (plastic 40%, sand 20%, and iron 40%) had optimum strength of 54.52 MPa, which was 1.54 times higher than OPC mortar. The SEM image in Figure 10 indicated that the particles of sand are angular and coarser but of uniform size, which required higher plastic to fill the voids completely. There is no perfect bond between sand particles and PET plastic. Sand is non-cohesive, so the particle can easily disintegrate into small fragments during the compression test.

### 3.4. Flexural Strength of PET Mortar having Marble and Iron Slag

**Figure 12:**
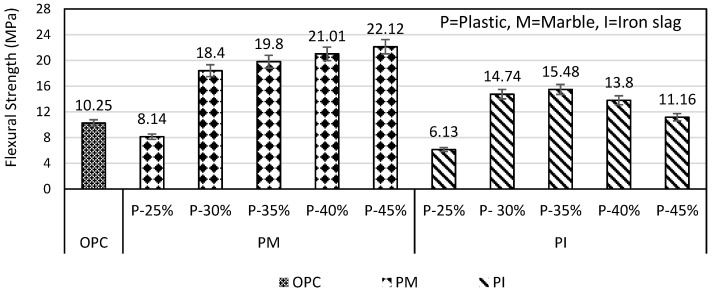
PM and PI specimen flexural strength.

The flexural strength of PM specimens increased from 18.4 to 22.12 MPa (i.e., 20.21%) when plastic content increased from 30 to 45%, as shown in Figure 12. The reason for enhanced strength was the fine particles of marble which require more plastic to bind it completely. In the case of the PI specimen, when plastic increased from 30 to 35%, flexural strength increased from 14.74 to 15.48 MPa, which was 5.02%, but when plastic further increased to 45%, the flexural strength decreased to 11.16 MPa which was 27.90% as shown in Figure 12. The strength of the PM mortar at every percentage of plastic was higher than the PI mortar. PM and PI mortar with plastic ranging from 30 to 45% possessed higher flexural strength than that of OPC. The optimum flexural strength of PM and PI specimens achieved at 45 and 35% plastic content, respectively, was 2.15 and 1.50 times higher than that of OPC, respectively. 

### 3.5. Effect of Sand Substitution on Flexural Response of PET Mortar

**Figure 13:**
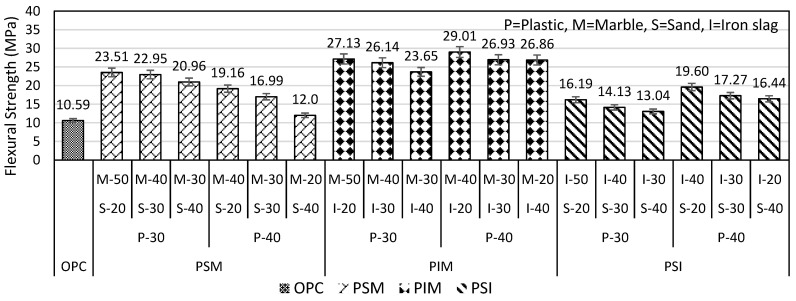
Flexural strength of plastic mortar (with 3 ingredients).

The results of the flexural strength of the three ingredients are shown in Figure 13. PSM-P30% and PSM-P40% flexural strengths were reduced from 23.51 to 20.96 MPa (i.e., 10.84%) and from 19.16 to 12.0 MPa (i.e., 37.36%), respectively, when sand content increased from 20 to 40%. Similarly, the flexural strength of PSI-P30% and PSI-P40% decreased from 16.19 to 13.04 MPa (i.e., 19.45%) and from 19.60 to 16.44 MPa (i.e., 16.12%), respectively, when increasing sand from 20 to 40%, as shown in Figure 13.

On the other hand, the PIM specimen was considered the best mix because the optimum strength of 29.01 MPa was 2.83 times higher than OPC mortar. The optimum strength of the PIM specimen was achieved at a mix ratio of (plastic 40%, iron slag 20%, and marble powder 40%). PIM-P30 and PIM-P40 specimens’ strength decreases from 27.13 to 23.65 MPa (i.e., 12.82%) and from 29.01 to 26.86 MPa (i.e., 7.4%), respectively, when iron slag increased from 20 to 40%, as shown in Figure 13. The results of the PSM, PIM, and PSI plastic mortar showed that every mix design of plastic mortar had a flexural strength higher than that of OPC mortar. 

### 3.6. Effect of Aggressive Chemicals Environment on PET Mortar

The durability of plastic mortar in terms of mass loss of plastic marble mortar (PM-P30 and PM-P40), plastic iron mortar (PI-P30 and PI-P40), and (PSM, PIM and PSI specimens with optimum strength) were studied. 

#### 3.6.1. Acidic Solution (5% HCl)

**Figure 14:**
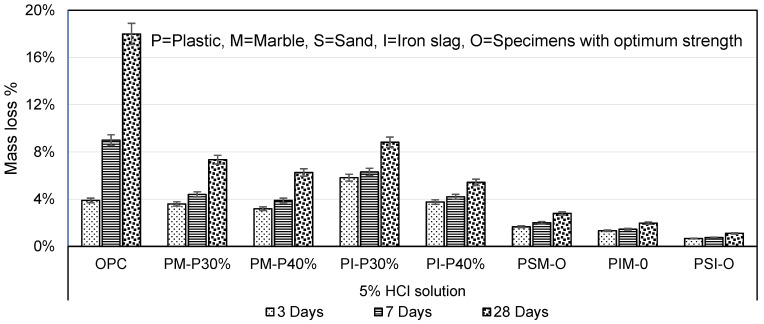
Mass loss in 5% HCl solution.

The durability of PM and PI specimens, when immersed in 5% HCl for 3, 7 and 28 days, showed a reduction in mass loss. The mass losses of PM-P30% and PM-P40% were 2.45 and 2.88 times, and PI-P30% and PI-P40% were 2.04 and 3.33 times less than OPC mortar, respectively, as shown in Figure 14. Another combination was PSM-O, PIM-O, and PSI-O specimens with optimum strength. The mass losses of PSM-O, PIM-O, and PSI-O were smaller than PM and PI specimens and were 6.45, 9.12 and 16.31 times less than OPC mortar, respectively. The reduction in mass losses was due to a reaction between hydrochloric acid (HCl) and marble powder, which is mainly composed of calcium carbonate (CaCO_3_) and represented by the following chemical equation [31]:

CaCO_3_ + 2HCl → CaCl_2_ + CO_2_ + H_2_O

In this reaction, the hydrochloric acid reacts with the calcium carbonate in the marble powder to produce calcium chloride (CaCl_2_), carbon dioxide (CO_2_), and water (H_2_O). The reaction is exothermic and releases heat. 

PM and PI specimen mass loss decreased with increasing plastic from 30 to 40%. The mass loss of PM and PI specimens decreased with increasing plastic content, which is in agreement with Benosman et al. (2011) study on PET mortar [40]. The PSI specimen was highly resistant to HCl acid solution among all other mix designs of mortar.

#### 3.6.2. Basic Solution (20% NaOH)

**Figure 15:**
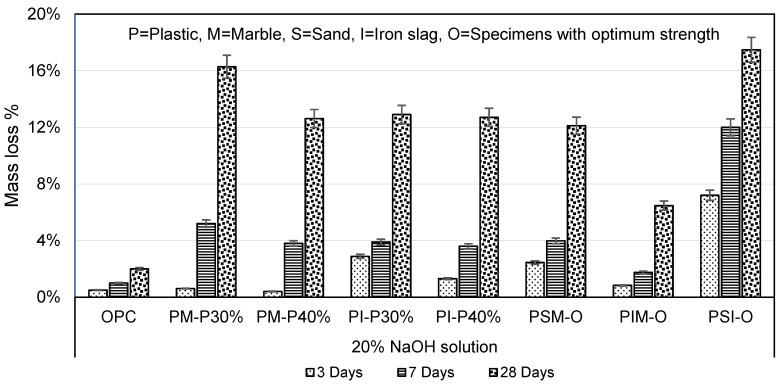
Mass loss in 5% NaOH solution.

**Figure 16:**
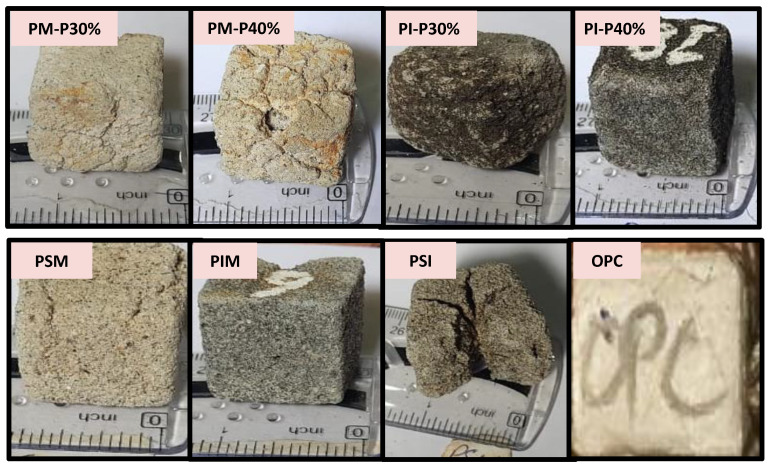
PET mortar after 28 days immersion in 20% NaOH solution.

The plastic mortar when placed in NaOH solution for 3, 7, and 28 days showed less resistance than acid and saline solution, as shown in Figure 15. PM-P30% and PM-P40% mass losses were 8.13 and 6.31 times, and PI-P30% and PI-P40% mass losses were 6.44 and 6.35 times higher than that of OPC mortar, respectively. The results showed that increasing plastic content in PM and PI specimens reduce mass losses in NaOH solution. Similarly, PSM-O, PIM-O, and PSI-O mass losses were 12.11, 3.23, and 8.73 times higher than that of OPC mortar, respectively. Figure 16 shows PET mortar specimens after 28 days of immersion in NaOH solution.

Marble, primarily composed of calcium carbonate, reacted with sodium hydroxide (NaOH) to form calcium hydroxide Ca (OH)_2_ and sodium carbonate (Na_2_CO_3_) Drechsler and Graham (2005) [41]. The reaction can be described by the following chemical equation:

CaCO_3_ + 2 NaOH → Ca (OH)_2_ + Na_2_CO_3_

Continuous exposure to NaOH can cause the marble to deteriorate and weaken, potentially leading to a reduction in its overall strength and durability. It is essential to carefully consider the use of NaOH in any environment where it may come into contact with marble. 

#### 3.6.3. Saline Solution (16% NaCl) 

**Figure 17:**
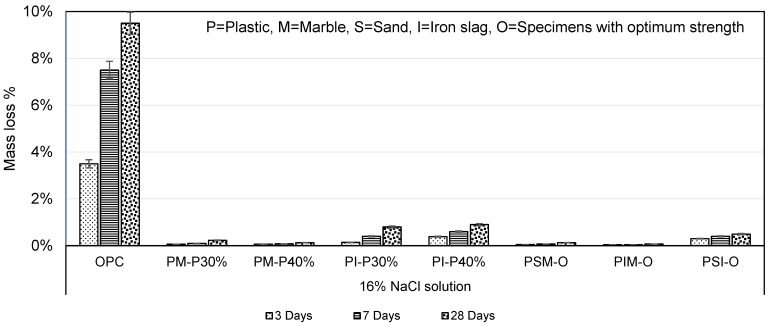
Mass loss in 16% NaCl solution.

Plastic mortar was found highly resistant to saline solution in terms of mass loss. The mass losses of PM-P30% and PM-P40% in 16% NaCl solution were 41.12 and 73.07 times less than OPC mortar, respectively, as shown in Figure 17. Althoey (2021) studied the effects of different NaCl concentrations at temperatures of 25 °C and 5 °C on cement paste and found that a deterioration occurred due to chemical changes in cement paste [42]. PI-P30% and PI-P40% mass losses were 11.87 and 10.55 times less than OPC mortar. The PET fibre-reinforced cement composite maintained its strength in NaCl solution, indicating excellent salt chemical resistance, as described by Won et al. (2010) [43]. PSM-O, PIM-O, and PSI-O mass losses were 72.51, 95, and 19.19 times less than OPC mortar, as shown in Figure 17. The mass loss results inferred that NaCl had a negligible effect on any of the ingredients used in plastic mortar.

### 3.7. Compressive Strength under Harsh Chemical Environment

**Figure 18:**
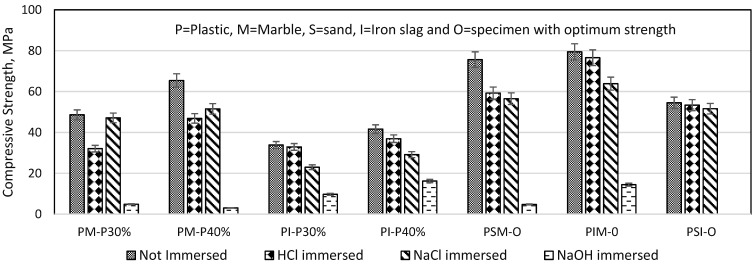
Compressive strength after 28 days in HCl, NaOH, and NaCl solutions.

After 28 days of immersion in aggressive solutions, the compressive strength of the plastic mortar was reduced. Figure 18 shows the reduction in compressive strength of chemically immersed plastic mortar. HCl and NaCl had less effect, while NaOH immersion caused a significant loss in compressive strength of plastic mortar. Humam and Siddique (2013) found that after 60 days in an alkaline environment, the strength of recycled PET fibre-reinforced cement composite deteriorated [39]. After 28 days in NaOH solution, PSI-O (PSI specimen with optimum strength) expand and crack, as shown in Figure 16. Thus, the compressive strength of the PSI-O specimen has diminished entirely. Won et al. (2010) found that NaOH can cause the marble to deteriorate and weaken, potentially leading to a reduction in the overall strength and durability [43]. 

### 3.8. Recycling Effects on Plastic Mortar Compressive Strength

**Figure 19:**
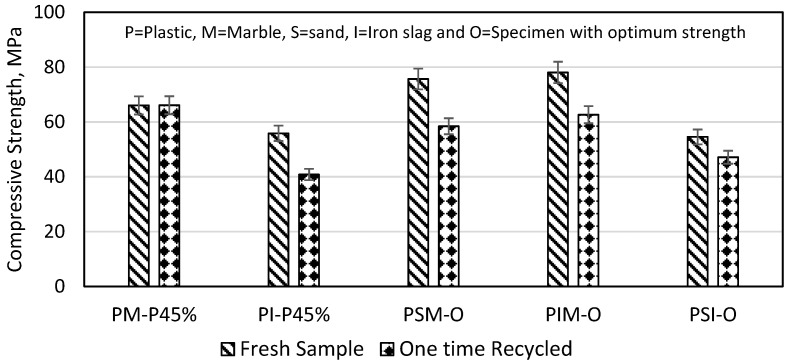
Comparison of fresh and recycled plastic mortar.

The recycling effect was evaluated via compressive strength tests on fresh and recycled plastic mortar specimens. The specimens were first subjected to a compressive strength test. The crushed sample obtained from compressive strength were remelted and poured into a mould that had been preheated to a temperature of 280 °C. The purpose of this was to study the effects of recycling on the compressive strength of the specimens. The results showed that the compressive strengths of the PM and PSM specimens were not affected by the recycling process, as shown in Figure 19. This indicates that these materials can be recycled without compromising their strength. However, the compressive strengths of the PI, PIM, and PSI specimens decreased by 26.87%, 19.74%, and 22.76%, respectively, after one-time recycling. This decrease in strength is due to the loss of plastic that occurs during the reheating process. The results revealed that iron slag and sand were more susceptible to plastic loss than marble powder. This is because iron slag and sand are coarser and poorly graded, which demands for higher plastic content for bonding compared to marble powder.

## 4. Conclusions

In this study, PET mortar was made by mixing melted waste PET bottles with marble powder, sand, and iron slag. The study results indicate that waste PET bottles can be used as a binder in mortar due to their good compressive and flexural strength. The PET mortar was also tested in various harsh chemical environments like 5% hydrochloric acid (HCl), 20% base (NaOH), and 16% salt (NaCl) solutions. The following are some findings of this study:

- The compressive and flexural strength of the PM specimen increased with increasing plastic content from 25 to 45%. The PI specimen’s compressive and flexural strength increased with increasing plastic content from 30 to 35% and reduced when plastic content further increased up to 45%. The optimum compressive strengths of PM, PI, PSM, PIM, and PSI specimens were 1.87, 1.24, 2.14, 2.25, and 1.54 times greater than that of the OPC mortar, respectively.
- In comparison to OPC mortar, all mixes of plastic mortar were more resistant to HCl and NaCl solution but less resistant to NaOH solution. Among different plastic mortar mixes, PIM mortar with optimum strength was relatively more resistant to NaOH and NaCl solutions. Though its mass loss in NaOH solution was still 3.23 times higher than that of the OPC mortar.
- Plastic mortar can be one-time recycled because the compressive strength degrades very negligibly after recycling. The decrease in compressive strength of one-time recycled plastic mortar containing marble was negligible, but a significant reduction in compressive strength was seen in the plastic mortar with iron slag.
- The compressive strength of plastic mortar decreased almost completely in a base solution but decreased less in an acid or saline solution. It is worth noting that PET mortar should be protected from NaOH base solution.

This study shows that PET mortar is susceptible to NaOH solution, and this limitation requires careful consideration for its industrial applications where a basic environment is expected. It is recommended to evaluate the permeability and thermal conductivity of both fresh and recycled PET mortar. The effect of temperature on mechanical properties and the determination of bonding strength is also recommended for future work. Additionally, this work is limited to the compressive strength evolution of one-time recycling PET mortar. Therefore, further investigation is recommended for the flexural strength and durability of recycled PET mortar.

## Figures and Tables

**Table 1:** Physical properties of aggregates.

Aggregate Type	Specific Gravity, G_s_ (ASTM D 854-14) [33]	Coefficient of Uniformity, C_u_ (ASTM D 6913) [32]	Coefficient of Curvature, C_c_ (ASTM D 6913) [32]
Sand	2.71	2.25	1.046
Marble Powder	2.66	3.72	1.09
Iron Slag	3.56	6.06	1.42

**Table 2:** Elemental composition of river sand, waste marble powder, and iron slag.

	River Sand	Marble Powder	Iron Slag
Element	Weight %	Weight %	Weight %
O	57.53	45.92	51.31
Mg	0.38	0.31	-
Al	8.7	-	5.26
Si	20.27	-	4.01
Na	10.05	-	-
K	0.42	-	0.33
Ca	0.28	32.87	0.46
Fe	1.42	0.21	10.84
C	-	20.69	27.79

**Table 3:** Specimens with two and three ingredients.

Specimens with Two Ingredients (by Weight)				
Type of Plastic Mortar	Mixture ID	Plastic %	Iron Slag %	Marble %
Plastic + Marble powder	PM-P25	25	-	75
	PM-P30	30	-	70
	PM-P35	35	-	65
	PM-P40	40	-	60
	PM-P45	45	-	55
Plastic + Iron slag	PI-P25	25	-	75
	PI-P30	30	-	70
	PI-P35	35	-	65
	PI-P40	40	-	60
	PI-P45	45	-	55
**Specimens with Three Ingredients (by Weight)**				
**Type of Plastic Mortar**	**Mixture ID**	**Sand %**	**Iron Slag %**	**Marble %**
Plastic 30% + sand and marble	PSM-P30	20	-	50
		30	-	40
		40	-	30
Plastic 40% + sand and marble	PSM-P40	20	-	40
		30	-	30
		40	-	20
Plastic 30% + iron slag and marble	PIM-P30	-	20	50
		-	30	40
		-	40	30
Plastic 40% + iron slag and marble	PIM-P40	-	20	40
		-	30	30
		-	40	20
Plastic 30% + sand and iron slag	PSI-P30	20	50	-
		30	40	-
		40	30	-
Plastic 40% + sand and iron slag	PSI-P40	20	40	-
		30	30	-
		40	20	-

## Data Availability

The data presented in this study are contained within the article.
